# Risk factors for urinary tract infection in infants with unexplained hyperbilirubinemia: a single center case-control study

**DOI:** 10.3389/fped.2024.1332052

**Published:** 2024-01-25

**Authors:** Ing Chen, Li-Sang Hsu, Cai-Sin Yao, Jenn-Tzong Chang, Hsiao-Ping Wang, Nai-Wen Fang

**Affiliations:** ^1^ Division of Pediatric Neonatology, Department of Pediatrics, Kaohsiung Veterans General Hospital, Kaohsiung, Taiwan; ^2^ Department of Business Management, National Sun Yat-Sen University, Kaohsiung, Taiwan; ^3^ Department of Medical Education and Research, Kaohsiung Veterans General Hospital, Kaohsiung, Taiwan; ^4^ Department of Pediatrics, Pingtung Veterans General Hospital, Pingtung, Taiwan; ^5^ Division of Pediatric Nephrology, Department of Pediatrics, Kaohsiung Veterans General Hospital, Kaohsiung, Taiwan

**Keywords:** neonatal hyperbilirubinemia, jaundice, neonatal sepsis, urinary tract infections, phototherapy

## Abstract

**Background:**

Urinary tract infection (UTI) is a potential cause of neonatal jaundice. Nevertheless, there remains a lack of consensus regarding appropriate screening practices for UTI in infants with hyperbilirubinemia. This study aimed to analyze a group of jaundiced infants to assess the prevalence of UTI, explore potential risk factors, and examine the impact of UTI on the course and severity of neonatal jaundice.

**Methods:**

This retrospective case-control study was conducted on 150 jaundiced infants (aged < 8 weeks) without a known etiology in the hyperbilirubinemia work-up. All subjects underwent phototherapy treatment and UTI screening by catheterization. They were then classified into UTI and non-UTI groups based on urine culture results, with a positive urine culture indicating the growth of ≥10,000 colony-forming units. The clinical characteristics and jaundice-related parameters of both groups were analyzed.

**Results:**

Among the 150 jaundiced patients, the prevalence of UTI was 29%. There was a significantly higher male predominance in the UTI group, and patients with UTI also had a significantly longer duration of hospitalization compared to those without UTI. Significant risk factors associated with UTI in jaundiced infants included male gender and a peak total bilirubin level higher than 18 mg/dl during hospitalization. The most common pathogens identified in urine culture were *Escherichia coli* (41.9%) and *Enterococcus faecalis* (30.2%).

**Conclusion:**

In cases of neonatal jaundice where the underlying cause is not evident, screening for UTI should be performed, particularly when associated risk factors or inadequate response to phototherapy is present.

## Introduction

Neonatal hyperbilirubinemia, characterized by a yellowish discoloration of the skin, sclera, and mucous membranes, is a commonly benign condition observed in newborns during the initial days of life, affecting at least 60% of full-term and 80% of preterm neonates (1, 2). It is a transitional process attributed to the turnover of fetal red blood cells and the limited conjugation of bilirubin by the newborn's immature liver, followed by a natural decline over the ensuing weeks (3). Nevertheless, approximately one in ten newborns are at risk of developing clinically significant hyperbilirubinemia, defined as an unconjugated bilirubin concentration that requires treatment with phototherapy that varies with postnatal age and cause of the condition, with most prevalent risk factors being prematurity, hemolytic disease, perinatal infection, and exclusive breastfeeding (2). It is also noteworthy that hyperbilirubinemia, in the absence of accompanying symptoms or signs, can be the sole clinical manifestation of urinary tract infection (UTI) in neonates (4, 5). Understanding this relationship is crucial for appropriate clinical management in infants presenting with jaundice. Currently, the American Academy of Pediatrics (AAP) guideline on the management of hyperbilirubinemia in newborn infants of 35 or more weeks of gestation notes the importance of considering UTI as one of the causes of neonatal direct hyperbilirubinemia that requires prompt treatment (6). On the other hand, the National Institute for Health and Care Excellence (NICE) guideline recommends urine culture collection in newborns with prolonged jaundice (7). However, infants with unexplained indirect pathological hyperbilirubinemia are not specifically addressed in these guidelines. Several studies have provided evidence to support unconjugated hyperbilirubinemia as a significant and potentially the first presentation UTI in newborns (5, 8–10). Despite these findings, there is currently no consensus on when to screen for UTI in infants with unexplained indirect pathological hyperbilirubinemia.

In an effort to identify predictive parameters that may alert clinicians to screen for UTI in infants with hyperbilirubinemia, this study aims to analyze a cohort of jaundiced infants to assess the prevalence of UTI, explore any potential risk factors, and examine the impact of UTI on the course and severity of neonatal jaundice, with the goal of providing insights for healthcare professionals engaged in the care of infants with jaundice.

## Methods

**Figure 1:**
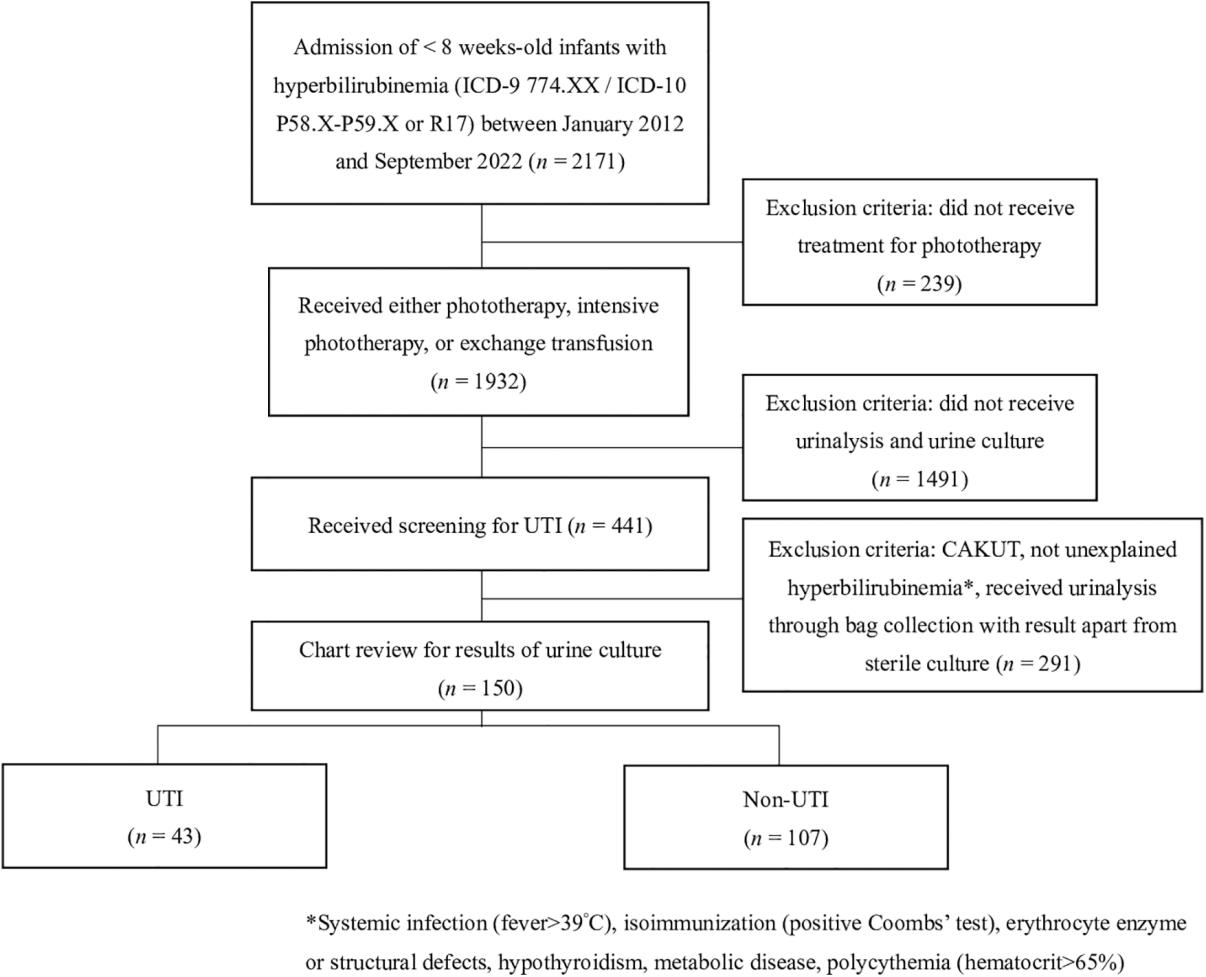
Flowchart of patient selection process. UTI, urinary tract infection; CAKUT, congenital anomalies of the kidney and urinary tract.

This case-control study was conducted by retrospectively extracting data from the clincal database of Kaohsiung Veterans General Hospital (KSVGH), Kaohsiung, Taiwan. Patients younger than 8 weeks of age who were admitted to KSVGH between January 2012 and December 2022 and met the diagnosis of neonatal jaundice, as identified by International Classification of Diseases, Ninth Revision (ICD-9) codes 774 or ICD-10 codes P58, P59, or R17, were enrolled, and their medical charts were subsequently reviewed. After detailed chart review, further excluded subjects include those who did not receive phototherapy treatment and screening for UTI, including urinalysis and urine culture. Patients with congenital anomalies of the kidney and urinary tract (CAKUT), known etiology in hyperbilirubinemia work-up, including systemic infection with fever >39°C, isoimmunization, erythrocyte enzyme or structural defects, hypothyroidism, metabolic disease, and polycythemia, were also excluded. Figure 1 illustrates the flowchart detailing the patient selection process.

Body weight and body length at admission, gender, gestational age, method of delivery, Apgar score, feeding method (exclusive breast feeding, formula feeding, or mixed feeding), maternal age, presence of maternal intrapartum fever, maternal Group B *Streptococcus* (GBS) screening status and whether intrapartum antibiotics prophylaxis (IAP) was used, and presence of premature rupture of membrane of more than 18 h were recorded. Prematurity was defined as a birth that occurs before 37 completed weeks of gestation. Onset of jaundice, total and direct serum bilirubin level at presentation, peak total serum bilirubin level during hospitalization, duration of phototherapy and requirement of intensive phototherapy or blood exchange transfusion, and duration of hospitalization were also recorded. In KSVGH, the treatment protocol for phototherapy is guided by the AAP and NICE guideline (6, 7). Our clinicians reference the Bhutani nomogram and utilize the treatment threshold graphs available on the NICE official website when prescribing phototherapy, intensive phototherapy, or exchange transfusion. Onset of jaundice was defined as the age of the patient at which phototherapy was initiated. Total bilirubin level was obtained by either capillary sample by heelstick or direct blood sampling. Response to phototherapy was derived based on decrease in serum total bilirubin at 24th and 48th hour of phototherapy and percentage decrease in serum total bilirubin at 24th and 48th hour of phototherapy. As part of hyperbilirubinemia and infectious survey, infants' and mothers' blood group, direct Coombs test, complete blood count, reticulocyte count, blood urea nitrogen, creatinine, C-reactive protein, blood culture were performed.

All the enrolled patients received screening for UTI including urinalysis and urine culture by catheterization. Pyuria was defined as urine white blood cell count (WBC) of ≥5 cells per high-power field (HPF) in the sediment of centrifuged urine (11). Microscopic hematuria was defined as presence of urine red blood cell count (RBC) of >5 cells/HPF (12). UTI was diagnosed based on positive urine culture from catheterized urine culture, defined as colony count ≥10,000 colony-forming units (13).

All statistical analyses were performed using SPSS for Windows, version 20 (SPSS Inc, Chicago, IL, USA). Continuous variables are presented as means with standard deviation and were compared using an independent samples *t-*test. Dichotomous variables were compared using the chi-squared test or Fisher's exact test. Univariate regression was performed, and variables with *P *< 0.1 were further assessed for their association with various factors using logistic regression. Statistical significance was set to *P *< 0.05.

This study was approved by the Institutional Review Board of KSVGH (KSVGH23-CT6-20).

## Results

A total of 2,171 infants younger than 8 weeks old were admitted to KSVGH under the diagnosis of neonatal jaundice, 150 of whom met the pre-specified inclusion criteria. Among these 150 patients, 43 (29%) were diagnosed with UTI. A summary of baseline demographic characteristics is presented in Table 1, showing no significant difference between the UTI and non-UTI groups aside from body weight at hospitalization and gender. UTI patients exhibited a significant higher male predominance and a higher body weight compared with non-UTI group.

**Table 1:** Demographic characteristics in jaundiced infants with and without UTI.

	Total	UTI	Without UTI	
Variables	*n* = 150	*n* = 43	*n* = 107	*P*-value
Body weight (gram)	3,003 ± 482	3,138 ± 560	2,949 ± 438	0.029
Body length (cm)	49.8 ± 2.2	50.4 ± 2.5	49.6 ± 2.1	0.052
Small for gestational age (*n*) (%)	7 (4.7%)	0 (0%)	7 (6.5%)	0.086
Prematurity (*n*) (%)	18 (12%)	8 (18.6%)	10 (9.4%)	0.115
Male (*n*) (%)	74 (49.3%)	32 (74.4%)	42 (39.3%)	<0.001
Cesarean section (*n*) (%)	55 (36.7%)	14 (32.6%)	41 (38.3%)	0.508
Apgar score at 1 min	7.95 ± 0.83 (*n* = 144)	8 ± 0.70 (*n* = 42)	7.93 ± 0.88 (*n* = 102)	0.654
Apgar score at 5 min	9.17 ± 0.83 (*n* = 144)	9.21 ± 0.52 (*n* = 42)	9.15 ± 0.95 (*n* = 102)	0.666
Risk of perinatal infection				
PROM >18 h (*n*) (%)	5 (3.3%)	1 (2.3%)	4 (3.7%)	0.663
Maternal positive GBS screening without adequate IAP (*n*) (%)	4 (2.7%)	1 (2.3%)	3 (2.8%)	0.869
Maternal intrapartum fever (*n*) (%)	2 (1.3%)	0 (0%)	2 (1.9%)	0.367
Feeding method	(*n* = 149)	(*n* = 43)	(*n* = 106)	0.900
Exclusive breast feeding (*n*) (%)	28 (18.8%)	8 (18.6%)	20 (18.9%)	
Formula feeding (*n*) (%)	45 (30.2%)	12 (27.9%)	33 (31.1%)	
Mixed feeding (*n*) (%)	76 (51%)	23 (53.5%)	53 (50%)	
Maternal age (years)	31.3 ± 5.5 (*n* = 131)	30.8 ± 5.7 (*n* = 40)	31.5 ± 5.4 (*n* = 91)	0.476

Data are given as mean ± standard deviation.

GBS, Group B *Streptococcus*; IAP, intrapartum antibiotics prophylaxis; PROM, premature rupture of membranes; UTI, urinary tract infection.

Outcomes in jaundiced infants with and without UTI are presented in Table 2. The mean time to onset of jaundice was 6.5 ± 6.2 days. The mean initial serum total bilirubin level was 17.4 ± 3.7 mg/dl, and only one patient in the overall study had conjugated hyperbilirubinemia. A total of 36 patients received intensive phototherapy. The mean duration of phototherapy was 3.8 ± 1.9 days, and the mean duration of hospitalization was 8.2 ± 4 days overall. The mean decrease in serum total bilirubin level at the 24th and 48th hour of phototherapy was 4 ± 3.1 mg/dl and 5.6 ± 4.2 mg/dl, respectively. The mean percentage decrease in serum total bilirubin at the 24th and 48th hour of phototherapy was 21.1 ± 19.3 percent and 29.7 ± 21.7 percent, respectively. Antibiotics were prescribed in 54 patients.

**Table 2:** Outcome in jaundiced infants with and without UTI.

	Total	UTI	Without UTI	
Variables	*n* = 150	*n* = 43	*n* = 107	*P*-value
Antibiotics prescribed (*n*) (%)	54 (36%)	34 (79.1%)	20 (18.7%)	<0.001
Onset of jaundice (day)	6.5 ± 6.2	7.5 ± 6.8	6.1 ± 6	0.200
Conjugated hyperbilirubinemia (*n*) (%)	1 (0.7%)	0 (0%)	1 (0.9%)	0.525
Initial serum total bilirubin (mg/dl)	17.4 ± 3.7	17.4 ± 2.7	17.2 ± 4	0.782
Decrease in serum total bilirubin (at 24th h) (mg/dl)	4 ± 3.1 (*n* = 130)	3.5 ± 2.7 (*n* = 36)	4.1 ± 3.3 (*n* = 94)	0.313
Percentage decrease in serum total bilirubin (at 24th h)	21.1 ± 19.3 (*n* = 130)	19.1 ± 14.1 (*n* = 36)	21.9 ± 20.1 (*n* = 94)	0.467
Decrease in serum total bilirubin (at 48th h) (mg/dl)	5.6 ± 4.2 (*n* = 124)	5.2 ± 4.5 (*n* = 35)	5.8 ± 4.1 (*n* = 89)	0.487
Percentage decrease in serum total bilirubin (at 48th h)	29.7 ± 21.7 (*n* = 124)	27.5 ± 25 (*n* = 35)	30.6 ± 20.4 (*n* = 89)	0.48
Peak total bilirubin level				
≥18 mg/dl	65 (43.3%)	24 (55.8%)	41 (38.3%)	0.051
<18 mg/dl	85 (56.7%)	19 (44.2%)	66 (61.7%)	
Intensive phototherapy (*n*) (%)	36 (24%)	7 (16.3%)	29 (27.1%)	0.160
Duration of phototherapy (days)	3.8 ± 1.9	3.8 ± 2.3	3.8 ± 1.8	0.907
Duration of hospitalization (days)	8.2 ± 4	11 ± 4.4	7 ± 3.2	<0.001
Serum				
WBC (1,000/ul)	12.7 ± 4.8	11.8 ± 3.7	13.1 ± 5.2	0.146
Neutrophil (%)	42.6 ± 15.5	40.9 ± 15.1	43.3 ± 15.6	0.379
Lymphocyte (%)	41 ± 15.3	44.4 ± 14.3	39.6 ± 15.5	0.079
Band (%)	1.7 ± 3	2 ± 2.4	1.6 ± 3.2	0.498
Hemoglobin (g/dl)	16.4 ± 2.4	15.9 ± 2.2	16.5 ± 2.5	0.188
Platelet (1,000/ul)	339.9 ± 112.8	344.2 ± 119	338.2 ± 110.7	0.770
BUN (mg/dl)	8.4 ± 4.2 (*n* = 112)	8.5 ± 4.1 (*n* = 32)	8.4 ± 4.3 (*n* = 80)	0.871
Creatinine (mg/dl)	0.55 ± 0.2 (*n* = 125)	0.55 ± 0.12 (*n* = 36)	0.56 ± 0.22 (*n* = 89)	0.365
C-reactive protein (mg/dl)	0.34 ± 0.54 (*n* = 149)	0.24 ± 0.32 (*n* = 43)	0.38 ± 0.61 (*n* = 106)	0.136
Positive blood culture (*n*) (%)	5 (3.6%)	2 (4.9%)	3 (3.1%)	0.552
Pyuria (*n*) (%)	25 (16.7%)	3 (7%)	22 (20.6%)	0.044
Microscopic hematuria (*n*) (%)	22 (14.7%)	8 (18.6%)	14(13.2%)	0.387

Data are given as mean ± standard deviation.

BUN, blood urea nitrogen; GBS, Group B *Streptococcus*; UTI, urinary tract infection; WBC, white blood cells.

Patients with UTI had a significantly longer duration of hospitalization compared to those without, and they also had a significantly higher rate of antibiotics usage. Otherwise, there were no differences in infectious screening results between the two groups, including differential count of WBC, hemoglobin, platelet, BUN, creatinine, C-reactive protein, blood culture positivity, and microscopic hematuria. There were a total of 5 positive blood culture results, all of which cultured coagulase-negative staphylococci. However, based on the physician's clinical judgment and subsequent blood culture assessments showing negative bacterial growth, contamination was favored. There was also no disparity between the UTI and non-UTI groups regarding the onset of jaundice, initial serum total bilirubin level, the number of cases receiving intensive phototherapy, duration of phototherapy, or response to phototherapy, as depicted by the decrease and percentage decrease in serum total bilirubin level.

Univariate logistic regression results indicated that body weight at admission (odds ratio [OR] = 2.19, 95% confidence interval [CI] = 1.05–4.57; *P* = 0.035), male gender (OR = 4.5, 95% CI = 2.04–9.89; *P* = <0.001), and antibiotics use (OR = 16.43, 95% CI = 6.81–39.66; *P* = <0.001) were independent risk factors for UTI in jaundiced infants. After adjusting for confounding factors through multivariate logistic regression analysis, the results, as shown in Table 3, revealed that a peak total bilirubin level higher than 18 mg/dl emerged as a risk factor for UTI [adjusted odds ratio (aOR) = 2.88, 95% CI = 1.08–7.64; *P* = 0.034]. Male gender remained a major determinant for UTI after multivariate analysis (aOR = 4.78, 95% CI = 1.72–13.24; *P* = 0.003), as did antibiotics use (aOR = 20.84, 95% CI = 7.57–57.36; *P* = <0.001). However, body weight at admission became insignificant during the multivariate logistic regression analysis.

**Table 3:** Multivariate logistic regression analysis for jaundiced infants with UTI.

Variable	aOR (95% CI)	*P*-value
Antibiotics prescribed	20.84 (7.57–57.36)	<0.001
Peak total bilirubin level ≥18 mg/dl	2.88 (1.08–7.64)	0.034
Gender, male	4.78 (1.2–13.24)	0.003
Body weight	1.07 (0.39–2.98)	0.894

aOR, adjusted odds ratio; CI, confidence interval; UTI, urinary tract infection.

**Figure 2:**
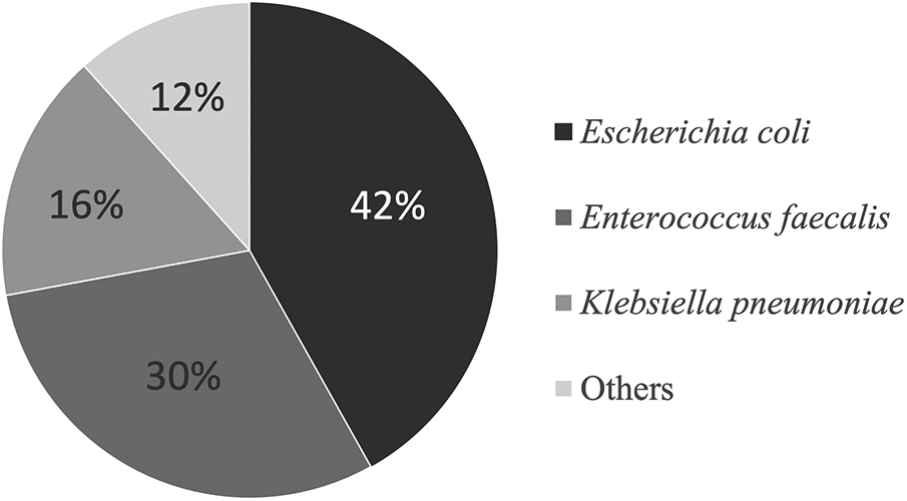
Microorganisms isolated by catheterized urine culture in jaundiced infants with UTI. UTI, urinary tract infection.

The most common microorganism identified in urine culture of UTI patients is *Escherichia coli* (41.9%), followed by *Enterococcus faecalis* (30.2%), and *Klebsiella pneumoniae* (16.3%), as depicted in Figure 2. We also analyzed the antibiotics sensitivity profile of the pathogens identified in the urine cultures, assessing the treatment success rates of various antibiotics against these pathogens. Among the commonly employed antibiotic regimens, the combination of ampicillin with gentamicin had a treatment success rate of 77%, The combination of ampicillin with a third-generation cephalosporin exhibited a treatment success rate of 72%, while the combination of cefazolin with gentamicin showed a rate of 60%. Notably, all isolated *Enterococcus spp.* in our urine samples exhibited sensitivity to ampicillin. Out of the total 43 patients diagnosed with UTI, 27 (61%) were evaluated by renal ultrasonography during hospitalization. Most patients had normal findings (63%), while others had mild hydronephrosis (29%), bilateral small kidneys (4%), and bladder wall thickening (4%).

In addition, the 150 patients enrolled in this study were divided into two groups based on whether antibiotics were prescribed during hospitalization, with clinical characteristics shown in Table 4. Significantly more patients were treated with antibiotics if they were diagnosed with UTI, had premature rupture of membranes for more than 18 h, or had leukocytosis, neutrophil predominance, or a higher level of C-reactive protein. Although the initial serum total bilirubin level was similar across the two groups, there was a significant difference between the two groups regarding parameters related to the response to phototherapy. Patients who received antibiotics treatment were significantly less responsive to phototherapy, showing a smaller decrease in serum total bilirubin level at the 24th and 48th h of phototherapy compared to patients who did not receive antibiotics treatment (2.9 ± 2.8 mg/dl vs. 4.6 ± 3.2 mg/dl at 24th h, and 4.5 ± 4.4 mg/dl vs. 6.2 ± 4 mg/dl at 48th h). Consistent results were also observed in the mean decrease in serum total bilirubin at the 24th and 48th h of phototherapy (15.7 ± 15.5 percent vs. 24 ± 20.6 percentage decrease at 24th h, and 24 ± 24.1 percent vs. 32.9 ± 19.8 percentage decrease at 48th h). Understandably, patients who received antibiotics treatment had a significantly longer length of stay for hospitalization.

**Table 4:** Clinical characteristics for antibiotics usage in jaundiced infants.

	Treated with antibiotics	Not treated with antibiotics	
Variables	*n* = 54	*n* = 96	*P*-value
With UTI (*n*) (%)	34 (63%)	9 (9.4%)	<0.001
Risk of perinatal infection			
PROM >18 h (*n*) (%)	4 (7.4%)	1 (1%)	0.037
Maternal positive GBS screening without adequate IAP (*n*) (%)	3 (5.6%)	1 (1%)	0.100
Maternal intrapartum fever (*n*) (%)	1 (1.9%)	1 (1%)	0.678
Onset of jaundice (day)	5.7 ± 5.1	6.9 ± 6.8	0.254
Initial serum total bilirubin (mg/dl)	16.7 ± 3.1	17.5 ± 4	0.179
Decrease in serum total bilirubin (at 24th h) (mg/dl)	2.9 ± 2.8 (*n* = 46)	4.6 ± 3.2 (*n* = 84)	0.003
Percentage decrease in serum total bilirubin (at 24th h)	15.7 ± 15.5 (*n* = 46)	24 ± 20.6 (*n* = 84)	0.018
Decrease in serum total bilirubin (at 48th h) (mg/dl)	4.5 ± 4.4 (*n* = 44)	6.2 ± 4 (*n* = 80)	0.028
Percentage decrease in serum total bilirubin (at 48th h)	24 ± 24.1 (*n* = 44)	32.9 ± 19.8 (*n* = 80)	0.030
Intensive phototherapy (*n*) (%)	11 (20.4%)	25 (26%)	0.435
Serum			
WBC (1,000/ul)	14.1 ± 5.6	12 ± 4.2	0.010
Neutrophil (%)	46.9 ± 14.9	40.2 ± 15.4	0.010
Lymphocyte (%)	38.6 ± 14.3	42.3 ± 15.7	0.158
Band (%)	2.3 ± 2.5	1.4 ± 3.1	0.080
Platelet (1,000/ul)	326.6 ± 112.5	347.4 ± 112.9	0.280
C-reactive protein (mg/dl)	0.47 ± 0.81 (*n* = 54)	0.27 ± 0.29 (*n* = 95)	0.030
Positive blood culture (*n*) (%)	2 (3.7%)	3 (3.1%)	0.850
Urine			
Pyuria (*n*) (%)	8 (14.8%)	17 (17.7%)	0.648
Microscopic hematuria (*n*) (%)	10 (18.5%)	12 (12.5%)	0.317

Data are given as mean ± standard deviation.

BUN, blood urea nitrogen; GBS, Group B *Streptococcus*; IAP, intrapartum antibiotics prophylaxis; PROM, premature rupture of membranes; UTI, urinary tract infection; WBC, white blood cells.

## Discussion

This study established that peak total serum bilirubin level higher than 18 mg/dl during serial follow-up during hospitalization is a risk factor for UTI in jaundiced patients. This aligns with the results of a few previous studies. One study examined newborns with early neonatal jaundice requiring phototherapy and found that those with concurrent diagnosis of UTI had significantly higher bilirubin levels at admission and at the 24th h of phototherapy compared to those without UTI. Additionally, the UTI group had a lower percentage decrease in bilirubin levels at the 24th h of phototherapy, indicating a poorer response to phototherapy (14). A recent study by Lo et al. investigated the risk of sepsis or UTI in well-appearing infants with jaundice under 7 days old and found that those with higher initial bilirubin levels had more abnormal C-reactive protein levels and a higher proportion of pyuria, although the rate of positive urine cultures did not differ (15). Ozdogan et al. evaluated jaundiced neonates 2–14 days old and found that those with concurrent UTI had significantly higher rebound bilirubin levels compared to those without UTI (9). A similar result was found in a study conducted by Mutlu et al., with a longer duration of phototherapy and higher rebound bilirubin level in neonates with UTI (16). The findings of a recent study conducted by Harb et al. revealed that, in jaundiced infants with and without UTI, a higher bilirubin level at admission and the highest peak of indirect bilirubin level were positively associated with presence of UTI (10). While our study did not replicate these exact findings, a similar trend was observed. Our study provided evidence that infants with jaundice who were also diagnosed with UTI tended to have more severe hyperbilirubinemia, as indicated by a higher peak total serum bilirubin level during serial follow-up. In addition, when comparing the response to phototherapy between jaundiced infants with and without UTIs, the UTI group had lower decreases and mean percentage decreases in bilirubin levels at the 24th and 48th h of phototherapy, although the differences were not statistically significant. Integrating the findings of previous studies with our own research, it can be inferred that infants diagnosed with both jaundice and UTI may exhibit a less favorable response to phototherapy and higher levels of serum bilirubin, despite receiving phototherapy (9, 10, 15, 16). Therefore, it is crucial to consider screening for UTI in jaundiced infants who show a poor response to phototherapy or have elevated levels of serum bilirubin.

To date, the precise mechanism behind the association between UTI and hyperbilirubinemia remains elusive, but there are several hypotheses. One possible explanation is that microorganisms associated with UTI or their circulating endotoxins may cause hepatocellular injury, leading to neonatal jaundice (8). Other proposed mechanisms include hemolysis, impaired bilirubin conjugation, and reduced elimination through the intestines caused by neonatal infections (16). Because of the immature conjugation mechanisms in infants, even slight hemolysis caused by *E. coli* and other Gram-negative microorganisms can lead to indirect hyperbilirubinemia (4).

Previous studies have examined the risk of UTI in patients with neonatal jaundice. The reported incidence of UTI in neonates with unexplained indirect hyperbilirubinemia ranged from 11% to 21.1% (5, 8, 9). The prevalence of UTI in jaundiced infants younger than 8 weeks old with an unknown etiology in this study is 29%, which is higher than the rates reported in previous studies. However, this disparity may be due to differences in local epidemiology or differences in UTI screening policies between institutions. At KSVGH, screening for UTI was routinely performed in jaundiced neonates admitted via the emergency department or outpatient clinic. Additionally, in line with the guidelines recommended by NICE and AAP, urine collection was also conducted in those with prolonged or direct hyperbilirubinemia. Jaundiced neonates who did not undergo routine UTI screening at our institution included individuals whose initial admission diagnosis was unrelated to hyperbilirubinemia. In these patients, who were already under treatment for their primary condition, hyperbilirubinemia emerged as a secondary issue during their admission, and it was left to the attending physician's clinical judgment whether to perform UTI screening. While not all neonatal jaundice cases at our hospital underwent UTI screening, potentially leading to selection bias, our screening policies ensure that those who did receive UTI screening were exclusively patients whose primary diagnosis was unexplained neonatal jaundice without other possible confounding factors, such as already being under treatment for another condition. This could account for why the prevalence of UTI in our study is higher compared to past literature. A study by Chen et al. investigated afebrile, asymptomatic newborns admitted due to neonatal jaundice at Changhua Christian Children's Hospital, Taiwan, and found the rate of positive urine culture was 14.3% (17). However, even though the local epidemiology is expected to be comparable to our study, Chen et al. did not specifically exclude jaundiced patients with clear etiology, which could potentially lower the prevalence of UTI in their study. Overall, the prevalence of UTI in infants with unexplained indirect hyperbilirubinemia is relatively high, underscoring the importance of evaluating for UTI in this population. For comparison, the reported rate of UTI in febrile neonates younger than 30 days is 15.4% (18), making the presentation of jaundice as important as fever when diagnosing neonatal UTI (4).

A meta-analysis of newborns with unexplained hyperbilirubinemia conducted in Iran showed that the risk of UTI is increased approximately two times in males and patients with low birth weight, compared with female gender and normal birth weight (19). However, a few studies found no significant difference in the risk of UTI between the two genders (5, 8, 20). The male predominance of UTI in our study is consistent with the meta-analysis by Amiri et al. and other studies, an expected result as the male gender is known to account for 75% of neonates and young infants with UTI (14, 18, 21–23). Yet, the present study differs from the one by Amiri et al. in that, instead of patients with low birth weight being a risk factor for UTI in jaundiced newborns, there was no association between body weight and UTI after multivariate analysis.

In our study, the prevailing causative pathogen for UTI is *Escherichia coli*, which accounts for 41.9% of total microorganisms identified in urine cultures. This is in concordance with previous studies and the general microbiologic data of febrile neonatal UTI (8, 9, 16, 18–20, 24). In our research, the second and third most prevalent pathogens associated with UTI were *Enterococcus faecalis* and *Klebsiella pneumoniae*. It is worth noting that while the prevalence of identified microorganisms in urine cultures may vary across studies, *Klebsiella spp.* and *Enterococcus spp.* consistently emerge as among the more frequently encountered species (8, 9, 16, 20). In our data collection, we studied the antibiotics sensitivity profile of the uropathogens identified in the urine cultures of the UTI cases, aiming to provide insights into antibiotics recommendation for cases of jaundiced infants with UTI. Out of the commonly employed antibiotics regimen, the combination of ampicillin with gentamicin had the highest treatment success rate at 77%, surpassing the combination of cefazolin with gentamicin, which achieved a success rate of only 60%. Therefore, the combination of ampicillin and gentamicin could be recommended as a promising choice for antibiotics therapy in jaundiced infants with UTI, which aligns with current recommendation regarding antibiotics therapy for neonatal UTI and febrile young infant in general (25). However, clinician should also take into consideration local surveillance of pathogens and antibiotics susceptibility patterns when choosing appropriate initial antibiotics treatment.

This study also examined the clinical characteristics associated with antibiotic usage in jaundiced infants. This analysis aimed to identify parameters that could assist clinicians in guiding the appropriate use of antibiotics, considering that jaundice can sometimes be a manifestation of neonatal sepsis (26). Other than the obvious risk factors, including elevated inflammatory markers and increased risk for perinatal infection, poor response to phototherapy is also associated with increased use of antibiotics. The association that clinicians are more inclined to prescribe antibiotics in patient with a poor response to phototherapy could imply a relationship between neonatal infection and response to phototherapy; however, this assumption is constrained by the limited sample size and data of our study, and it necessitates further evidence.

The strengths of this study lie in further solidifying the importance of screening for UTI in patients with neonatal jaundice, especially if the patient has poor response to phototherapy and elevated peak bilirubin level despite treatment, with a cut-off value of 18 mg/dl. Secondly, the study design offers high validity in UTI diagnosis in that all the urine samples were obtained by sterile catheterization documenting growth of >10,000 of the same microorganism. Thirdly, as the database was obtained from a tertiary medical center in Taiwan, the result could be extrapolated to provide local assessment of UTI prevalence and risk factors in Taiwan. This study was limited in that not all patients who were diagnosed with UTI had imaging studies and long-term follow-up. Also, this was only a tertiary single-center study, which sets limit to the sample size.

## Conclusion

In conclusion, our study demonstrated that infants with jaundice who also had UTI tended to have more severe hyperbilirubinemia, as indicated by a higher peak total serum bilirubin level during serial follow-up. Additionally, male gender emerged as a risk factor for UTI in jaundiced infants. Our investigation also identified the most common uropathogens in this patient group, providing insights for recommending antibiotic regimens based on the sensitivity tests of urine cultures. In view of our findings, we suggest screening patients with pathological jaundice without a clear etiology for possible UTI, especially if risk factors or poor treatment response is present.

## Data Availability

The raw data supporting the conclusions of this article will be made available by the authors, without undue reservation.
